# The quaternary lidocaine derivative QX-314 in combination with bupivacaine for long-lasting nerve block: Efficacy, toxicity, and the optimal formulation in rats

**DOI:** 10.1371/journal.pone.0174421

**Published:** 2017-03-23

**Authors:** Qinqin Yin, Jun Li, Qingshan Zheng, Xiaolin Yang, Rong Lv, Longxiang Ma, Jin Liu, Tao Zhu, Wensheng Zhang

**Affiliations:** 1 Laboratory of Anesthesia and Critical Care Medicine, Translational Neuroscience Center, West China Hospital, Sichuan University, Chengdu, Sichuan, P. R. China; 2 North Sichuan Medical College, Nanchong, Sichuan, P. R. China; 3 Center for Drug Clinical Research, Shanghai University of Chinese Medicine, Shanghai, P. R. China; 4 Kunming Medical University, Kunming, Yunnan, P. R. China; 5 Department of Anesthesiology, West China Hospital, Sichuan University, Chengdu, Sichuan, P. R. China; University of Bari, ITALY

## Abstract

**Objective:**

The quaternary lidocaine derivative (QX-314) in combination with bupivacaine can produce long-lasting nerve blocks *in vivo*, indicating potential clinical application. The aim of the study was to investigate the efficacy, safety, and the optimal formulation of this combination.

**Methods:**

QX-314 and bupivacaine at different concentration ratios were injected in the vicinity of the sciatic nerve in rats; bupivacaine and saline served as controls (n = 6~10). Rats were inspected for durations of effective sensory and motor nerve blocks, systemic adverse effects, and histological changes of local tissues. Mathematical models were established to reveal drug-interaction, concentration-effect relationships, and the optimal ratio of QX-314 to bupivacaine.

**Results:**

0.2~1.5% QX-314 with 0.03~0.5% bupivacaine produced 5.8~23.8 h of effective nerve block; while 0.5% bupivacaine alone was effective for 4 h. No systemic side effects were observed; local tissue reactions were similar to those caused by 0.5% bupivacaine if QX-314 were used < 1.2%. The weighted modification model was successfully established, which revealed that QX-314 was the main active ingredient while bupivacaine was the synergist. The formulation, 0.9% QX-314 plus 0.5% bupivacaine, resulted in 10.1 ± 0.8 h of effective sensory and motor nerve blocks.

**Conclusion:**

The combination of QX-314 and bupivacaine facilitated prolonged sciatic nerve block in rats with a satisfactory safety profile, maximizing the duration of nerve block without clinically important systemic and local tissue toxicity. It may emerge as an alternative approach to post-operative pain treatment.

## Introduction

More than 200 million people undergo invasive procedures or surgeries every year all around the world [1]. Most of them need postoperative pain management. Commonly used narcotic analgesics (opioids) and non-steroid anti-inflammation drugs (NSAIDs) can cause systemic adverse effects such as vomit, nausea, respiratory depression, gastro-intestinal bleeding, and renal function impairment. Comparatively speaking, local anesthetics provide pain relief in a safe way. However, the action of traditional local anesthetics seldom lasts over 8 h in adults [2], while the intense postoperative pain usually exists for 1–3 days [3]. Developing long-lasting local anesthetics, therefore, is in great needs.

Different from classic local anesthetics, the N-ethylated derivative of lidocaine (QX-314) is positive charged under physiological conditions, therefore difficult to penetrate through cell membranes to reach the action sites: sodium-channels on the cyptoplasm of neurons. However, it can enter neurons through Transient Receptor Potential Ankyrin 1 (TRPA1) and Transient Receptor Potential Vanilloid 1 (TRPV1) channels that are activated by capsaicin [4], protons [5], and local anesthetics [6] [7], to produce prolonged local anesthesia. Among commonly used local anesthetics, bupivacaine is the most potent one in activating TRPA1 channels. Co-application of 0.5% bupivacaine and 0.5% QX-314 produced prolonged sciatic nerve block in rats, suggesting potential clinical values.

However, bupivacaine, like other amide amino local anesthetics, is concentration-dependent toxic to nerve and muscle tissues [8]. QX-314 also demonstrates local tissue toxicity when applied at high concentrations [9]. Accidental intravascular injection of local anesthetics can cause central nervous system toxicity (such as unconsciousness, seizures, or coma) and cardiovascular toxicity (such as bradycardia, arrhythmia, hypotension, and cardiovascular collapse). In particular, the cardiac arrest induced by bupivacaine can be refractory. Cheung *et al*. reported that QX-314 is more systemically toxic than lidocaine [10]. It is possible that the combination of QX-314 and bupivacaine could lead to synergism in local or systemic toxicity. Under this circumstance, the possibility of clinical application of this combination will depend on whether the benefits of prolonged nerve blockades could outweigh the risks of toxicities.

To address this issue, detailed concentration-effect relationships, particularly, the appropriate concentration ratio between QX-314 and bupivacaine that could maximize the duration of peripheral nerve blockade without clinically important systemic toxicities and tissue injuries will be needed.

We therefore investigate: 1) duration of effective nerve block, systemic adverse effects, and local tissue reactions produced by peri-sciatic nerve injection of different formulations; and 2) the optimal formulation based on efficacy and safety.

## Materials and methods

### Drug preparation

0.75% Bupivacaine hydrochloride solution (Jiang Su Heng Rui Medicine Co., Ltd., Lianyungang, China) was diluted with saline (Qingshan Likang Pharmaceutical, Co., Ltd., Chengdu, China) to obtain different concentrations needed. QX-314 as white crystalline power was purchased from Sigma Alderich (Shanghai, China), and was dissolved in bupivacaine hydrochloride solutions as required. The QX-314 powder quickly dissolves in the solution of bupivacaine, because QX-314 is highly hydrophilic. The solution was vibrated for 2 min to ensure that it is fully dissolved.

Bupivacaine at 1 mmol/L (0.03%), 3 mmol/L (0.1%), 5 mmol/L (0.2%), 10 mmol/L (0.3%), and 15 mmol/L (0.5%) were used, according to clinical applications. For QX-314, 5 mmol/L (0.2%) is the lowest concentration reported to produce anti-nociception with lidocaine, but was ineffective when used alone [6]; 45 mmol/L (1.5%) QX-314 is the highest concentration with normal gross appearance of local tissues after peri-sciatic application [9]; 25 mmol/L (0.9%) is at the lower inflection point of the dose-effect curve of QX-314, and the minimum concentration required to produce nerve blocks with normal tissue morphology [9], [11]. The concentrations of QX-314 used in combinations with bupivacaine were 5 mmol/L (0.2%), 10 mmol/L (0.3%), 15 mmol/L (0.5%), 20 mmol/L (0.7%), 25 mmol/L (0.9%), 35 mmol/L (1.2%), and 45 mmol/L (1.5%).

Thirteen combinations, namely 5 mmol/L QX-314 with 5 mmol/L bupivacaine (Q5B5), 10 mmol/L QX-314 with 10 mmol/L bupivacaine (Q10B10), 15 mmol/L QX-314 with 3 mmol/L bupivacaine (Q15B3) or 10 mmol/L bupivacaine (Q15B10) or 15 mmol/L bupivacaine (Q15B15), 20 mmol/L QX-314 with 5 mmol/L bupivacaine (Q20B5) or 10 mmol/L bupivacaine (Q20B10) or 15 mmol/L bupivacaine (Q20B15), 25 mmol/L QX-314 with 5 mmol/L bupivacaine (Q25B5) or 10 mmol/L bupivacaine (Q25B10) or 15 mmol/L bupivacaine (Q25B15), 35 mmol/L QX-314 with 1 mmol/L bupivacaine (Q35B1), and 45 mmol/L QX-314 with 10 mmol/L bupivacaine (Q45B10), were freshly prepared in the morning of experimental days, with 15 mmol/L bupivacaine (B15) served as the positive control and 0.9% sodium chloride (Saline) served as the negative control. Test solutions were prepared freshly within 1 h before the animal experiments. The final pH of solutions containing bupivacaine and QX-314 was 5.5 to 6.5 (S40 Sevenmulti^™^ pH meter, USA).

### Animal care

The animal experiments were performed according to the National Research Council's guidelines, and approved by the Committee of Scientific Research and Institutional Animal Experimental Ethics, West China Hospital, Sichuan University (Approval file No. 2015014A). Invasive procedures were conducted under isoflurane anesthesia, and efforts were made to minimize animal suffering. All animal experiments were carried out in accordance with the guide for the care and use of medical laboratory animals (Ministry of Health, China). Male Sprague-Dowley rats (Dossy Experimental Animal Company, Chengdu, China), aged 3~6 months, weighted 274 ± 46 g (250~367 g), were housed at room temperature in the 12-h light/12-h dark cycle with free access to food and water. The rats were acclimated to experimental environment.

### Sciatic nerve block procedures

Animals received peri-sciatic nerve injection under inhalation anesthesia with 1.5~2.0% isoflurane (Yipin Pharmaceutical, Shijiazhuang, China) [11]. A 29-Gauge needle was introduced at the one-third distance of the imaginary line connecting the greater trochanter and ischial tuberosity (caudal to the greater trochanter). 0.2 mL of test solution was injected once the tip of the needle encountered the ischium.

### Assessment of nerve blockade

The thermal nociceptive thresholds and muscle strength were used to evaluate sensory and motor function, respectively. The revised hot plate test was performed to assess the thermal nociceptive thresholds. Briefly, a rat was gently restrained by a towel and the paw of the injected limb was placed on a 56°C metal plate (RB-200 Hot Plate, Chengdu Techman Software Co. Ltd.). The time for a rat to withdraw its paw (paw withdrawal latency, PWL), reflecting the degree of inhibition of thermal sensation, was measured. The PWL measured before the formal experiments (baseline) was 1.9 ± 0.2 s. A cut off time of 12 s was used to avoid tissue injury; PWL exceeded 7 s was considered effective sensory blockade, for it is the halfway between cutoff time of 12 s and baseline of 2 s [11].

To measure the muscle strength, the extensor postural thrust test was employed, in which the hind paw was placed against an electric balance (HZT-5000, Fuzhou Huazhi Technology Co. Ltd.). The value (in grams) displayed by the balance represented the force that the legs could exert. Muscle strength, measured by extensor postural thrust test, was 150 ± 20 g before peri-sciatic injection. A decrease in this value is proportional to the degree of the suppression of motor function. The percentage of decrease in this value from baseline was calculated, and 50% of decrease was defined as effective motor blockade [11].

The behavioral tests were conducted by a researcher who was blinded to the treatments rats received.

### Evaluation of toxicity

Rats were observed for systemic adverse effects including sensory and motor function deficits of the untreated limbs, sedation, convulsion, seizure, ataxia, excitation, loss of weight, and death [10]. Animals were also inspected for local side effects including skin rashes, edema, muscle spasm and self-mutilation throughout the 2-week observation period after injection.

Histological examinations were performed at the end of observation period. Animals were euthanized by intra-peritoneal injection of overdose pentobarbital. The sciatic nerves with the surrounding tissues were harvested. Hematoxylin-eosin staining (HE-staining) was performed [12]. The morphological changes of tissues were analyzed by three pathologists using the BX51 microscope system (Olympus, Tokyo, Japan) in a blinded fashion. A 0–4 scale was used to semi-quantitatively evaluate the degree of inflammation, necrosis, degeneration and vacuolation within epineurium and in the adjacent muscles, where 0 = normal; 1 = 0~25% of area involved; 2 = 25%~50% of area involved; 3 = 50%~75% of area involved; and 4 = 75%~100% of area involved [13].

### Mathematical model establishment

For the QX-314 + bupivacaine combination, the weighted modification model (Formula 1) was used to investigate the relationship between the durations of nerve blockade and the following variables: the standardized concentration of each ingredient (*X*_*i*_, for QX-314 or bupivacaine, *i* = 1 or 2, respectively); the derivative variables including exponent (*X*_*i*_^*2*^) and drug-interaction (*X*_*12*_); and the randomization effects including the intragroup variation (*η*) and the residual error (*ε*). The standardized concentration is obtained by dividing the concentration applied of a drug with the mean value of all concentrations used. The derivative variables were added into the model if they caused decreased in objective function value (OFV) that is of statistical significance. (OFV > 3.84, Chi-square test, *df* = 1, *P* < 0.05).

$${\text{E}}_{\text{obs}}=\frac{{\text{E}}_{\text{max}}}{\gamma }\cdot \frac{{\text{B}}_{1}{\text{X}}_{1}+{\text{B}}_{2}{\text{X}}_{2}+{\text{B}}_{3}{\text{X}}_{1}^{2}+{\text{B}}_{4}{\text{X}}_{2}^{2}+{\text{B}}_{12}{\text{X}}_{1}{\text{X}}_{2}}{{\text{X}}_{50}+{\text{B}}_{1}{\text{X}}_{1}+{\text{B}}_{2}{\text{X}}_{2}+{\text{B}}_{3}{\text{X}}_{1}^{2}+{\text{B}}_{4}{\text{X}}_{2}^{2}+{\text{B}}_{12}{\text{X}}_{1}{\text{X}}_{2}}+\eta \;+\;\varepsilon$$ (1)

Molar concentration (mmol/L) instead of weight/volume percentage concentration (w/v %) was used for the sake of the accuracy and conveninency in calculation. *E*_*max*_ is the maximal effect, namely the longest duration of nerve block achieved in animal experiments. *γ* is the degree of flatness of the dose-effect curve. *X*_*50*_ is the standardized concentration corresponding to the half of *E*_*max*_. *η* followes the normal distribution of *N* (0, ω^2^). *ε* followes the normal distribution of *N* (0, σ^2^). *B* is the weight index of variables. A drug with a higher value of weight index is the main active component in the combination; *B*_*12*_ > 0 suggests synergism; *B*_*12*_ < 0 suggests antagonism; and *B*_*12*_ = 0 indicated no interaction effect. Diagnostic plots, including goodness-of-fit and distribution of residues, were used to evaluate the final models.

Modeling and simulation were conducted with DAS 4.0 (version 2.1, Bioguider Medicinal Technology Co. Ltd., Shanghai, China). Model parameters were obtained by the nonlinear mixed effect method.

### Statistical analysis

The Shapiro-Wilk test was used to test whether the data could be analyzed by the parametric method. Duration of the nerve blockade conformed to an approximating normal distribution and were expressed as mean ± SD. In our preliminary experiments, significant difference was revealed with sample size of six, and alpha set at 0.01. Sample sizes in formal experiments were set at a minimum of six rats, based on the preliminary experiments.

Levene test was performed to detect the homogenity of variances. Because both sensory and motor nerve blockades had unequial variances, non-parameter comparison using Kruskal-Wallis test was employed to analysis the difference of nerve blockades between treatmemnts. The histological scores were skewly distributed, and they were compared by the Kruskal-Wallis H test followed by the pair-wise Mann Whitney U-test. The *F*-test was used for the final model evaluation. The *χ*^*2*^ test was conducted for adding the derivative items. The statical analysis on the original data was conducted by SPSS version 21.0 (IBM, USA). Difference was considered significant if *P*< 0.05.

## Results

### Durations of effective nerve blockades

Saline was ineffective; 0.5% bupivacaine provided 3.6 ± 1.3 h of effective sensory blockade and 4.0 ± 0.8 h of motor blockade. Kruskal-Wallis test revealed significant difference among groups (for sensory and motor nerve blockade, *P* < 0.001). Three formulatons, namely Q25B15, Q35B1, and Q45B10, produced sensory (*P* = 0.012, *P* = 0.001, and *P* < 0.001, respectively) and motor blockade (*P* = 0.018, *P* = 0.009, and *P* = 0.001, respectively) that were statistically longer than 0.5% bupivacaine (Table 1).

**Table 1:** Duration of nerve blockades from QX-314 in combination with bupivacaine.

Combinations		Abbreviation	*n*	Sensory duration, h	Motor duration, h
QX-314, mmol/L (w/v %)	Bupi, mmol/L (w/v %)				
5 (0.2)	5 (0.2)	Q5B5	6	4.2 ± 1.9	4.0 ± 1.1
10 (0.3)	10 (0.3)	Q10B10	10	6.0 ± 2.2	5.2 ± 1.9
15 (0.5)	3 (0.1)	Q15B3	6	5.8 ± 3.4	5.1 ± 2.2
15 (0.5)	10 (0.3)	Q15B10	10	5.8 ± 2.1	5.6 ± 1.8
15 (0.5)	15 (0.5)	Q15B15	8	5.2 ± 3.0	3.8 ± 1.3
20 (0.7)	5 (0.2)	Q20B5	10	5.8 ± 1.6	5.8 ± 1.6
20 (0.7)	10 (0.3)	Q20B10	8	7.3 ± 2.3	6.3 ± 3.3
20 (0.7)	15 (0.5)	Q20Q15	8	6.8 ± 3.0	6.1 ± 3.1
25 (0.9)	5 (0.2)	Q25Q5	10	8.6 ± 6.1	6.2 ± 2.5
25 (0.9)	10 (0.3)	Q25B10	8	9.0 ± 3.0	5.7 ± 2.3
25 (0.9)	15 (0.5)	Q25B15	6	10.1 ± 0.8*	10.1 ± 0.8*
35 (1.2)	1 (0.03)	Q35B1	6	15.2 ± 6.8**	14.5 ± 7.3**
45 (1.5)	10 (0.3)	Q45B10	6	23.8 ± 0***	23.8 ± 0**
15 mmol/L (0.5%) Bupi		B15	8	3.6 ± 1.3	4.0 ± 0.8
Saline		Saline	6	0	0

Bupi: bupivacaine.

* *P* < 0.05 *vs*. 0.5% Bupivacaine;

** *P* < 0.01 *vs*. 0.5% Bupivacaine;

*** *P* < 0.001 *vs*. 0.5% Bupivacaine.

Kruskal-Wallis test demonstrated statistical difference among the thirteen combinations (S1 Data. *P* < 0.001). Moreover, when the concentration of QX-314 was fixed, the duration of sensory nerve blockade did not statistically differ as the concentration of bupivacaine changed (*P* > 0.05 for QX-314 equal to 15, 20, and 25 mmol/L). On the contrary, when the concentration of bupivacaine was fixed, altering the concentration of QX-314 resulted statistically different duration of sensory nerve blockade (*P* = 0.046, *P* < 0.001, and *P* = 0.015 for bupivacaine equal to 5, 10, and 15 mmol/L). The tendency of QX-314 being the main active ingredient for sensory blockade was largely replicated for motor blockade. Maintaining the concentration of bupivacaine at 10 and 15 mmol/L but increasing the concentration of QX-314 resulted statistically longer lasting motor blockade (*P* = 0.003 and *P* = 0.002 respectively). The duration of motor blockade did not statistically differ from each other for QX-314 equal to 15 and 20 mmol/L (*P* > 0.05).

### Toxicities

**Fig 1:**
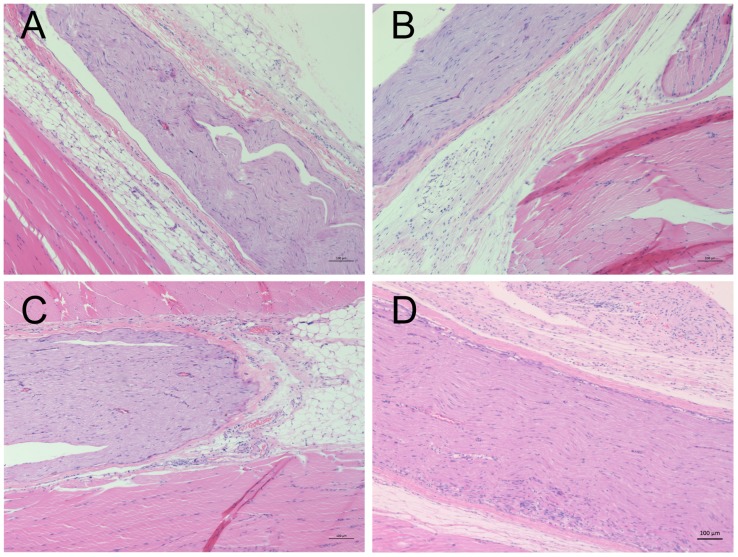
Representitive images of histological apperance two weeks after sciatic nerve blocks. (A): Saline; (B): 0.5% bupivacaine; (C): 1.2% QX-314 + 0.03% bupivacaine; and (D): 1.5% QX-314 + 0.3% bupivacaine. Moderate to severe granulocytes infiltration, edema, degeneration, and vacuolization were frequently observed in combination where QX-314 was used ≥ 1.2%.

Behaviors indicating systemic toxicity or acute local irritation were absent. Bupivacaine with 1.2% or 1.5% QX-314 induced moderate to severe inflammatory cell inflitration in muscles and nerve tissues (Fig 1), despite that thermal thresholds and muscle strength in all rats returned to the values before the injections. Correspondingly, the histological scores of muscle inflammation for 1.2% QX-314 + 0.03% bupivacaine (median score 1.5, ranged 0 to 3) and 1.5% QX-314 + 0.3% bupivacaine (median score 2.5, range 1 to 3) were significantly higher than that for 0.5% bupivacaine (median score 0, ranged 0 to 1). Similarily, the scores of inflammation of nerve tissues for 1.2% QX-314 + 0.03% bupivacaine (median score 0, ranged 0 to 1) and 1.5% QX-314 + 0.3% bupivacaine (median score 0.5, range 0 to 2) were higher than that for 0.5% bupivacaine (median score 0, ranged 0 to 0). There was no statistical difference in histological scores between 0.5% bupivacaine and the rest combinations (*P* > 0.05).

### Concentration-effect relationships, drug interaction, and the optimal combination

**Fig 2:**
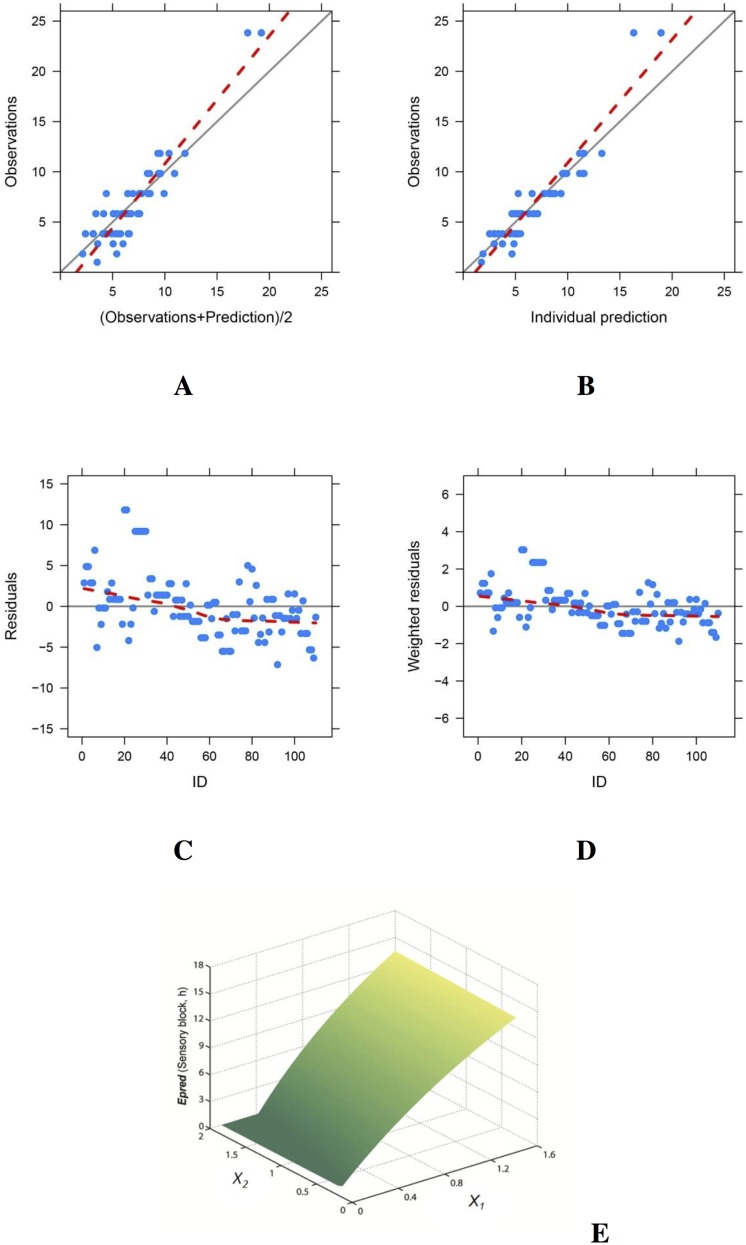
The final weighted modification model for sensory blocks. The model predictions were in reasonable agreement with the observations (A, B). Individual residuals were evenly distributed (C); and the weighted residuals were within ± 4 (D). The response-surfaces for sensory block (E) indicated that the duration of effective nerve blockade (*Epred*) prolonged as the concentration of QX-314 (*X*_*1*_), but not bupivacaine (*X*_*2*_), increased.

**Fig 3:**
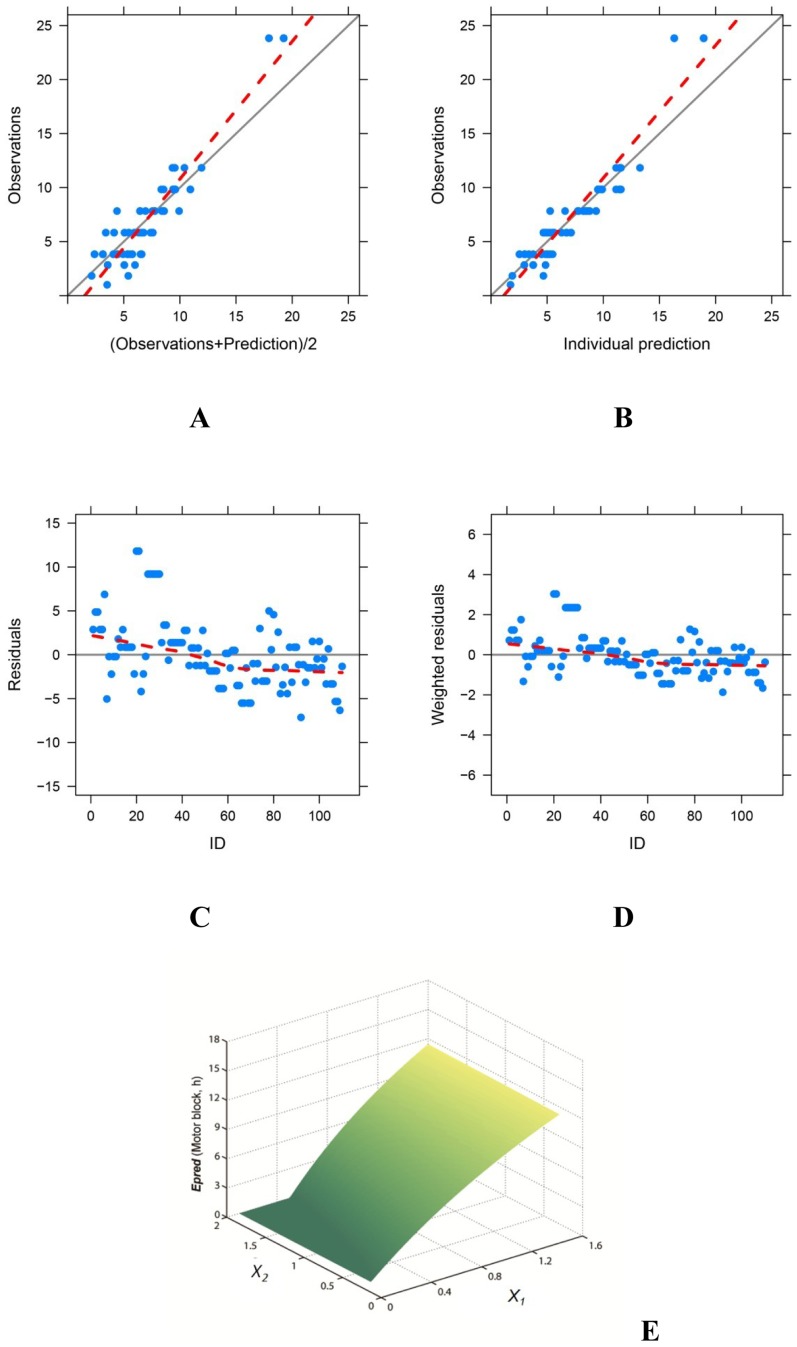
The final weighted modification model for motor blocks. The model predictions were in good agreement with the observations (A, B). Individual residuals were evenly distributed (C); and the weighted residuals were within ± 4 (D). The response-surfaces for motor block (E) indicated that the duration of effective motor blockade (*Epred*) prolonged as the concentration of QX-314 (*X*_*1*_), but not bupivacaine (*X*_*2*_), increased.

Weighted modification models were established successfully (*P* < 0.001, Figs 2A–2D and 3A–3D) with the two types of parameters (Table 2) which indicated synergism between the two component (*B*_*12*_ > 0); QX-314 was the main active ingredient while bupivacaine was the synergist in both sensory and motor blocks. Duration of nerve block prolonged as the concentration of QX-314 increased; but bupivacaine has limited impact on extending nerve blocks (Figs 2E and 3E).

**Table 2:** Parameters in the final models.

Parameters	Sensory block		Motor block		Meaning
	Value	SD	Value	SD	
*B*_*1*_	0.874	0.410	0.008	0.004	The weight index of QX-314
*B*_*2*_	-0.260	0.124	-0.003	0.002	The weight index of bupivacaine
*B*_*12*_	0.187	0.171	0.002	0.001	The weight index of drug-interaction
*Emax/γ*	40.910	2.748	33.944	2.298	The maximal effect/the flatness of the dose-effect curve
*X50*	3.004	1. 417	0.026	0.014	The concentration to achieve half *E*_*max*_
*η*	1.777	0.545	2.429	0.601	Covariate between groups
*ε*	1.984	0.187	1.872	0.172	Residual error

The model simulation revealed that the longest duration of both sensory (16.9 h) and motor (14.9 h) block were achieved by 45 mmol/L (1.5%) QX-314 plus 10 mmol/L (0.3%) bupivacaine. This concentration ratio also produced the longest action of nerve block (23.8 h) in animal experiments, consistent with the model prediction. But considering there were severe tissue inflammation when QX-314 was used ≥ 1.2% (35 mmol/L), the concenration of QX-314 should be limited to ensure local tissue safety. Consequently, the optimal formulation with acceptable safety profile and the longest duration of nerve blockade should be 0.9% (25 mmol/L) QX-314 versus 0.5% (15 mmol/L) bupivacaine. It produced sensory block for 10.1 h and motor block for 9.0 h in model prediction, consistent with those demonstrated in animal experiments (10.1 ± 0.8 h).

## Discussion

The key of clinical translation of the combination of QX-314 and bupivacaine are the duration of anesthetic effects and local tissue injury. This study demonstrated that the effects of nerve blocks could be maximized with satisfactory safety profiles when 0.9% QX-314 and 0.5% bupivacaine were co-applied. The duration of effective sensory blockade resulting from the optimal combination was about 2.5-fold of that produced by 0.5% bupivacaine alone (10.1 ± 0.8 h *vs*. 3.6 ±1.3 h, *P* < 0.001), and there was no behavioral evidence of systemic toxicity or histological changes indicating local tissue injury. 15 mmo/L (0.5%) bupivacaine was chosen as the positive control because it is the highest concentration routinely used for nerve block.

Common methods to facilitate prolonged regional analgesia are continuous infusion of local anesthetics, controlled release local anesthetic formulations, and co-injection of local anesthetics with additives. Local tissue compatibility is the major limitation for controlled release local anesthetic formulations [14], [15], [16], [17]. The only controlled release local anesthetic formulation approved by FDA is Exparel^®^, liposomal bupivacaine. It is used exclusively in wound infiltrations [18], and its efficacy in nerve blocks is limited. Its magnitude in femoral nerve blocks was highly variable among healthy volunteers [19]; a recent phase 3 clinical trial using Exparel^®^ for intercostal nerve block after posterolateral thoracotomy resulted in an unsatisfactory reduction of cumulative pain scores. Continuous infusion of local anesthetics through indwelling catheters has the risks of mechanical injury and infections. Co-application of local anesthetic with additives is relatively convenient, cost-effective, and less toxic, but the effects of prolongation are limited using common additives such as dexamethasone, naloxone, clonidine, buprenorphine, and dexmedetomidine [20], [21], [22], [23], [24], [25], [26], [27]. Recently, ultra-long lasting nerve blocks have been achieved in rats with traditional local anesthetics in combination with two newly developed sodium channel blockers: neo-STX and EN3427. However, there are concerns about systemic and local tissue toxicity for these combinations. Localized myopathy and neuropathy were caused by high dose of EN3427, necessitating fully investigation of local tissue toxicity [28]. Peri-sciatic nerve application of neo-STX + bupivacaine led to transient sensory and motor function deficits of the untreated limb, sometimes gasping respiration and apnea in rats [29], which may indicate systemic toxicity with clinical significance under this route of administration.

This study revealed the safety profile of the combinations of QX-314 and bupivacaine in peri-sciatic administration. Bupivacaine, as well as QX-314, have inner activity to produce local neurotoxicity and myotoxicity, which is dose-dependent [9], [30]. Co-application of bupivacaine and QX-314 may further complicate local reactions. In this study, there were no sensory or motor nerve function deficits in the untreated limbs in all formulations, suggesting none or insignificant systemic distribution [12], [29]. Systemic administration of QX-314 has higher central nervous system and cardiac toxicities than lidocaine [10]. In this study, rats received peri-neural co-administration of QX-314 and bupivacaine did not develop any behavioral evidence of central nerve system or cardiac toxicities. Inflammations of local tissues were associated with high concentration of QX-314. However the degree of local tissue reactions to formulations in which QX-314 ≤ 25 mmol/L were minimal to mild, similar to 0.5% bupivacaine. The overall safety profile of QX-314 + bupivacaine combination was acceptable.

The duration of effective sciatic nerve blocks for 0.9% QX-314 + 0.3% bupivacaine was 10 h, 2.5 times longer than that by 0.5% bupivacaine. It should be noted that the sensory blockade was considered effective only when 50% suppression of nerve function was achieved (thermal threshold dropped halfway from cutoff value of 12 s to baseline of 2 s; or 50% decrease in the weight-bearing capacity). What we investigated was the duration of effective nerve blockade, instead of the time until fully recovery to baselines, which was longer than the former. It is the effective nerve block that would reduce pain scores and decrease the consumption of opioids.

Mathematical modeling is a quantitative method to describe the relationship between drug doses and therapeutic effects. The model-based drug development has been strongly recommended by FDA to aid efficient drug development [31]. Zheng *et al*. reported the weighted modification model for drug interaction analysis and dose optimization [32]. It was proved reliable in cases of assessing the optimal combination of allantoin, metronidazole, and dexamethasone for anti-inflammatory treatment [33], and dose optimization of irbesartan and hydrochlorothiazide for renal hypertension therapy [34]. The weighted modification model was used for the first time to analyze the interaction and to find the optimal formulation in regional anesthesia. It confirmed the synergism effect between QX-314 and bupivacaine, quantitatively assessed the importance of each component in producing therapeutic effects, and revealed the optimal concentration ratio. Interestingly, QX-314 was the main active component, whereas bupivacaine was the synergist in producing both sensory and motor blockade. The roles of QX-314 and bupivacaine in sensory nerve blockade can be explained by the theory that bupivacaine acts as a channel activator to promote cellular entry of QX-314. QX-314 is difficult to permeate through cell membranes. A high extracellular concentration of QX-314 (at least 0.9%) is required to elicit intracellular effects [6]. Brenneis *et al*. reported that bupivacaine activates TRPV1, TRPA1, and some unknown TRP-independent entry pathways expressed in neurons, which promote cellular entry of QX-314. However, since TRPV1 and TRPA1 are specifically distributed in nociceptive-related neurons, the unknown TPR-independent entry pathways that can be activated by bupivacaine to permit cellular uptake of QX-314 might exist in motor nerve fibers. This postulation warrants further electrophysiological studies.

Inevitably, this study has limitations. Firstly, we only investigated the sciatic nerve block. The efficacy and safety of the QX-314 + bupivacaine combination in other administration routes such as subcutaneous infiltration, epidural and intrathecal injection may differ from those in sciatic nerve blocks. Secondly, we did not explore the combination in inflamed tissues. Inflammation is one of the most common clinical problems, *e*.*g*. fractures, infections, and surgical wounds. In flamed tissues, TRPA1 is up-regulated, and QX-314 alone caused little change in paw withdrawal thresholds in inflamed mice [35]. Given that QX-314 is the main active ingredient, and its analgesia effects are at least partially mediated by TRPA1, the efficacy of the QX-314 + bupivacaine combination might be changed (possibly less efficient, because more QX-314 molecules might be required to block all the nerve fibers) in inflamed tissues. Thirdly, HE-staining is a widely-used method to assess local tissue toxicity, but is relatively insensitive to measure subtle nerve injury. Finally, species variation between rodents and human beings should be taken into consideration.

## Conclusion

0.9% QX-314 plus 0.5% bupivacaine provided long-lasting sciatic nerve blockade in rats with satisfactory safety profile. The QX-314 + bupivacaine combination may emerge as an alternative approach to post-operative pain treatment.

## Supporting information

S1 Data The original data. Duration of sciatic nerve blockade, and tissue histological scores.
(SAV)
