# Correction: The quaternary lidocaine derivative QX-314 in combination with bupivacaine for long-lasting nerve block: Efficacy, toxicity, and the optimal formulation in rats

**DOI:** 10.1371/journal.pone.0177203

**Published:** 2017-05-03

**Authors:** Qinqin Yin, Jun Li, Qingshan Zheng, Xiaolin Yang, Rong Lv, Longxiang Ma, Jin Liu, Tao Zhu, Wensheng Zhang

The image for Fig 2 is incorrect. Please see the complete, correct Fig 2 here.

**Fig 2:**
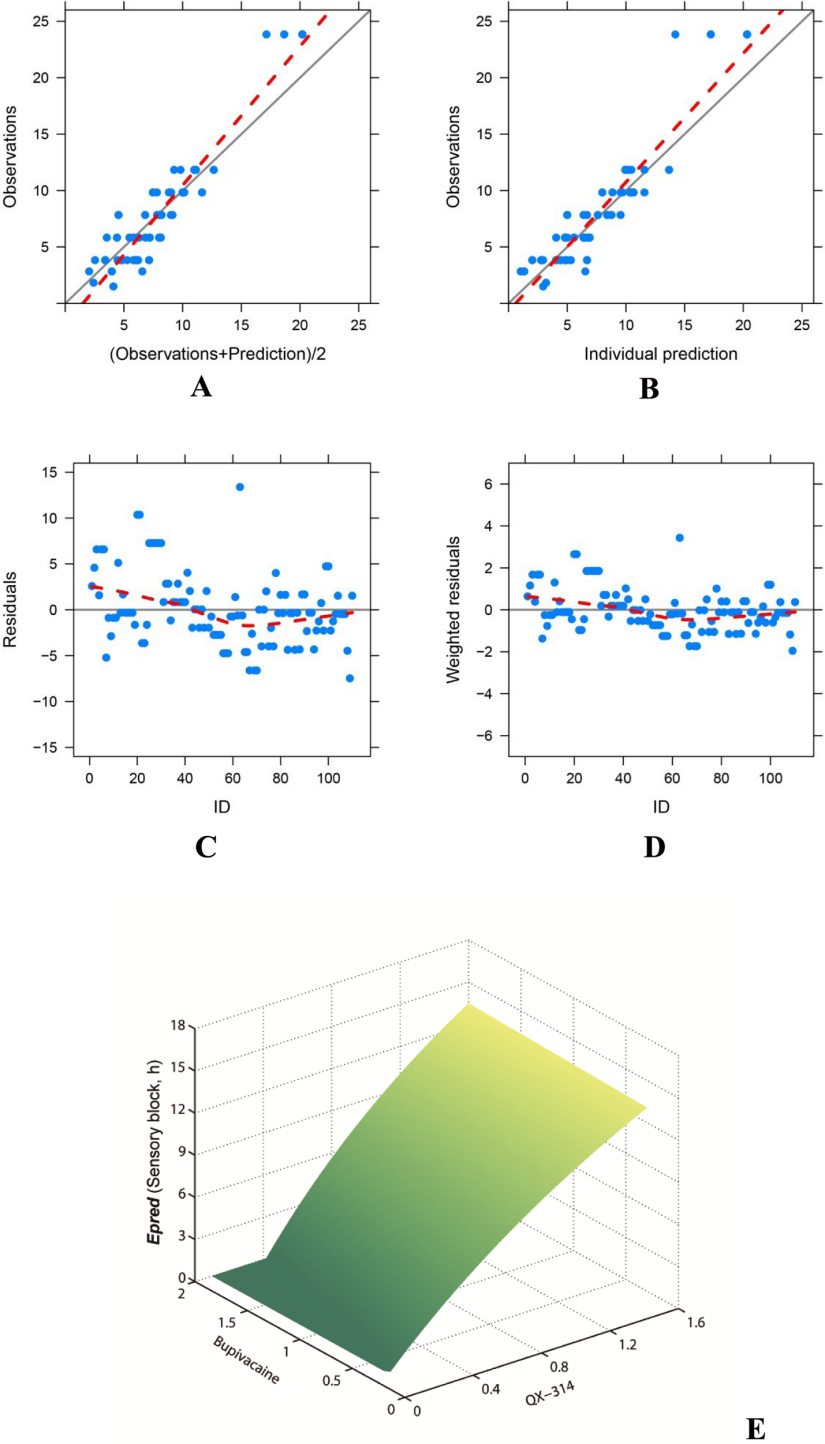
The final weighted modification model for sensory blocks. The model predictions were in reasonable agreement with the observations (A, B). Individual residuals were evenly distributed (C); and the weighted residuals were within ± 4 (D). The response-surfaces for sensory block (E) indicated that the duration of effective nerve blockade (*Epred*) prolonged as the concentration of QX-314 (*X*_*1*_), but not bupivacaine (*X*_*2*_), increased.
